# Book Notices

**Published:** 1891-09

**Authors:** 


### Book Notices.

MEDICAL SYMBOLISM in connection with Historical Studies in the Arts of Healing and Hygiene. Illustrated. By Thomas S. Sozinskey, M. D., Ph. D., author of "The Culture of Beauty," "The Care of Culture of Children," etc. Cloth: $1: pp. 171: F. A. Davis, publisher, Philadelphia and London, 1891.

This little book is a classical gem. It is short, and the writer has shown his good judgment in not burdening the pages with long quotations. Mythology in medicine boiled down to a readable volume is what the profession has expected for some time.

B.

FEVER: ITS PATHOLOGY AND TREATMENT BY ANTIPYRETICS. Being an essay which was awarded the Boylston prize of Harvard University, July, 1890. By Hobart Amory Hare, M. D., B. Sc., Philadelphia. pp. 162: price, $1.25 net. F. A. Davis, publisher, Philadelphia, 1891.

This book is No. 10 in the physicians' and students' ready reference series, and is a prize essay awarded by Harvard University. The latter is a sufficient guarantee of the merits of the work. B.

